# Real-World Clinical Outcomes of Darolutamide in Older Patients With Non-metastatic Castration-Resistant Prostate Cancer: A Multi-institutional Comparative Analysis of Efficacy and Tolerability

**DOI:** 10.7759/cureus.97911

**Published:** 2025-11-27

**Authors:** Yoichiro Tohi, Takuma Kato, Kengo Fujiwara, Yushi Hayashida, Yuki Matsuoka, Hiromi Hirama, Toshifumi Yano, Hiroyuki Tsunemori, Hironobu Arai, Mikio Sugimoto

**Affiliations:** 1 Department of Urology, Faculty of Medicine, Kagawa University, Takamatsu, JPN; 2 Department of Urology, Sakaide City Hospital, Sakaide, JPN; 3 Department of Urology, KKR Takamatsu Hospital, Takamatsu, JPN; 4 Department of Urology, Shodoshima Central Hospital, Shodoshima, JPN; 5 Department of Urology, Takinomiya General Hospital, Ayagawa, JPN; 6 Department of Urology, Mizushima Kyodo Hospital, Kurashiki, JPN

**Keywords:** age, darolutamide, elderly, non-metastatic castration-resistant prostate cancer, survival

## Abstract

Background

There is still a lack of real-world data comparing clinical use of darolutamide, oncological outcomes, and safety profiles among older patients. We aimed to evaluate the real-world clinical outcomes of darolutamide in older patients with non-metastatic castration-resistant prostate cancer (nmCRPC) by comparing efficacy and tolerability across different age groups.

Methods

We retrospectively analyzed 36 patients with nmCRPC who received darolutamide at nine institutions. Patients were stratified into two age-based subgroups: ≥75 vs. <75 years and ≥80 vs. <80 years. Primary outcomes were oncological, including prostate-specific antigen (PSA) response (50% or 90% decline), progression-free survival (PFS), and overall survival (OS). Secondary outcomes included safety, adverse events (AEs), and rates of treatment interruption, discontinuation, and dose reduction.

Results

Twenty-five patients (69.4%) were aged ≥75 years, and 13 patients (36.1%) were aged ≥80 years. The overall PSA response rates were 88.9% for a 50% decline and 69.4% for a 90% decline. PSA response did not differ between age groups for either 75- or 80-year thresholds. No significant differences were observed in PFS or OS between subgroups. The incidence of all-grade and grade ≥3 AEs was comparable across age groups. However, patients aged ≥80 years showed significantly higher rates of dose reduction or treatment interruption (P = 0.016).

Conclusions

Darolutamide demonstrated high PSA response rates and favorable survival outcomes in elderly patients with nmCRPC. Its effectiveness was consistent across age subgroups. While overall safety profiles were similar, patients aged ≥80 years were more likely to need dose adjustments or temporary treatment interruptions, supporting the use of this drug in this population.

## Introduction

Non-metastatic castration-resistant prostate cancer (nmCRPC) is characterized by increased prostate-specific antigen (PSA) levels despite castrate levels of testosterone, without radiologically detectable metastases on conventional imaging. Although patients are often asymptomatic at this stage, nmCRPC represents a critical transition phase with a high risk of progression to metastatic CRPC (mCRPC), which is associated with significantly shortened overall survival (OS) [1]. The advent of second-generation androgen receptor signaling inhibitors (ARSIs), such as darolutamide and enzalutamide, has transformed the treatment landscape for nmCRPC [2-5]. Darolutamide showed clinical efficacy in the phase III ARAMIS trial, significantly improving metastasis-free survival (MFS) and reducing the risk of death compared with placebo [2,3]. Darolutamide exhibits low blood-brain barrier penetration [6], which is associated with a low risk of central nervous system (CNS)-related adverse events (AEs), including seizures, falls, and cognitive impairment. This safety profile has made darolutamide a favorable option for patients for whom tolerability is a clinical priority [7].

A recent retrospective study showed that darolutamide was associated with lower rates of treatment discontinuation and mCRPC progression risks compared with enzalutamide or apalutamide in routine clinical practice, implying a potentially better tolerability profile [8]. Nevertheless, there is still a lack of real-world data directly comparing their clinical use, safety profiles, and impact on treatment adherence among diverse patient populations, especially elderly patients [9].

Considering these knowledge gaps, we conducted a multi-institutional real-world study in Japan with pre-specified age thresholds (≥75 and ≥80 years) to assess the efficacy and safety of darolutamide in elderly nmCRPC patients.

## Materials and methods

Ethics statements

This retrospective study received approval from the Institutional Review Board of Kagawa University (approval number: 2025-158). For the eight collaborating institutions that contributed only pre-existing samples and clinical data, a centralized ethical review was conducted at Kagawa University. All procedures complied with the ethical standards of the institutional and national research committees and adhered to the principles of the Declaration of Helsinki (1964) and its subsequent revisions. Owing to the retrospective nature of the study, the requirement for written informed consent was waived. However, study details were made publicly available on the institutional website, allowing patients to opt out if they did not wish to have their data included.

Patients' data

Data of patients treated with darolutamide for nmCRPC were retrospectively reviewed across nine Japanese institutions between September 2020 and August 2025.

Data collection

Patient demographic and clinical data were retrospectively collected from medical records, including age at darolutamide initiation, Eastern Cooperative Oncology Group (ECOG) performance status, height, weight, body mass index, PSA level at darolutamide initiation, Gleason grade at initial diagnosis, clinical T stage at initial diagnosis, prior ARSI use, history of radical treatment, PSA doubling time (PSA-DT) at darolutamide initiation, and AEs. Patients were divided into two age-based subgroups: ≥75 vs. <75 years and ≥80 vs. <80 years, and compared.

Study objective

Primary outcomes were oncological, including PSA response (≥50% or ≥90% decline from baseline), progression-free survival (PFS; defined by PSA increase or radiographic progression), and OS. Secondary outcomes were safety-focused, including AEs and rates of treatment interruption, discontinuation, and dose reduction. PSA response rates were calculated as the proportion of patients who achieve a ≥50% or ≥90% reduction in PSA levels from baseline. Radiographic progression was defined as the appearance of metastases on computed tomography or bone scintigraphy. Time to disease progression was defined as the interval from darolutamide initiation to PSA response or radiographic progression, in accordance with the Prostate Cancer Clinical Trials Working Group 2 (PCWG2) criteria [10], which was consistent with the previous study [11]. OS was defined as the interval from darolutamide initiation to death from any cause.

Statistical analysis

Quantitative variables were summarized as medians with interquartile ranges (IQRs). Comparisons of categorical variables between groups were performed using either the chi-square test or Fisher’s exact test, whereas continuous variables were analyzed with the Mann-Whitney U test. Time-to-event outcomes were evaluated using the Kaplan-Meier method and reported as medians with 95% confidence intervals (CIs) and corresponding P-values. Statistical significance was defined as P < 0.05. All statistical analyses were carried out using EZR (Saitama Medical Center, Jichi Medical University, Saitama, Japan), a graphical user interface for R (The R Foundation for Statistical Computing, Vienna, Austria) [12], and GraphPad Prism version 9.0.0 (GraphPad Software, San Diego, CA, USA).

## Results

Patient characteristics

**Figure 1:**
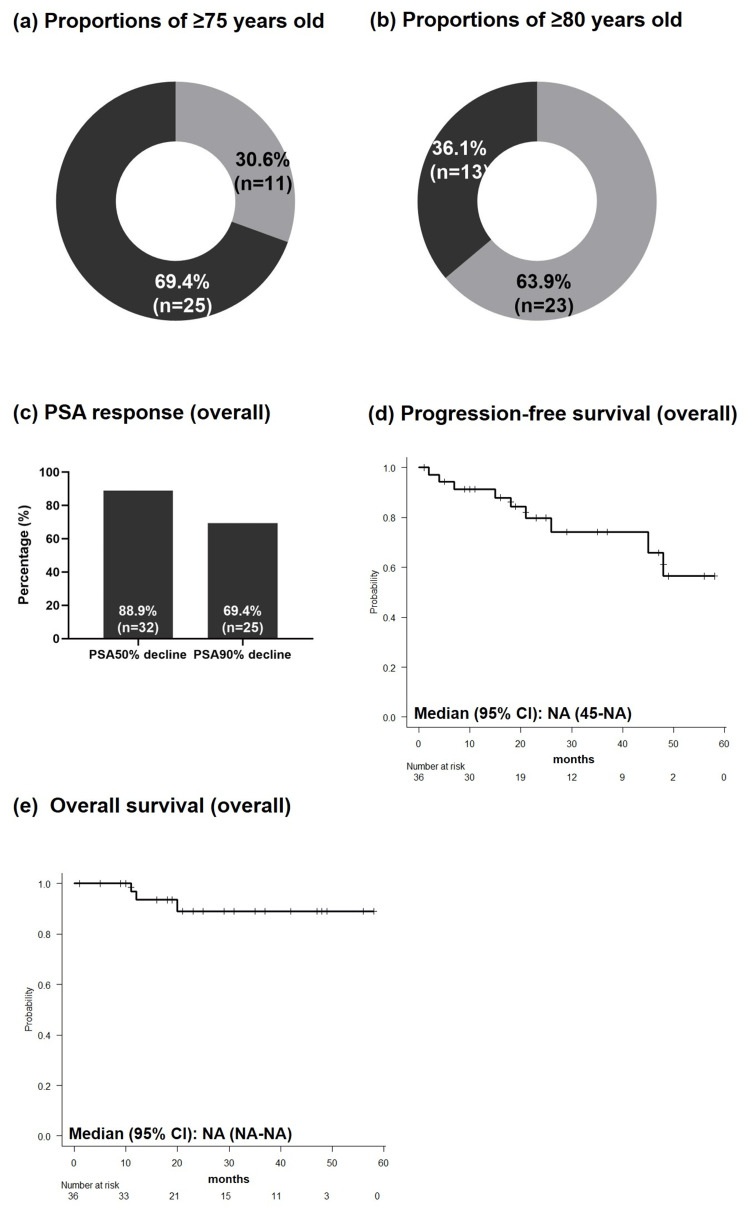
(a) Proportions of ≥75 years old, (b) proportions of ≥80 years old, (c) PSA response in the overall cohort, (d) progression-free survival in the overall cohort, (e) overall survival in the overall cohort NA: not available; PSA: prostate-specific antigen; CI: confidence interval

As shown in Table 1, 36 patients with nmCRPC were treated with darolutamide (median age 77.5 (IQR, 72.8-83.3) years). Of the patients, 25 (69.4%) were aged ≥75 years, and 13 (36.1%) were aged ≥80 years (Figures 1a, 1b). The median follow-up period from darolutamide initiation to last visit was 23.0 (16.0-47.3) months. Ten patients (27.8%) had received no prior radical treatment, 10 (27.8%) had undergone surgery, and 16 (44.4%) had undergone radiation therapy. Median PSA-DT at darolutamide initiation was 4.60 (3.0-7.1) ng/mL. Darolutamide was initiated at a dose of 1200 mg in 35 (97.2%) patients.

**Table 1:** Patients' characteristics IQR: interquartile range; ECOG PS: Eastern Cooperative Oncology Group performance status; ARSI: androgen receptor signaling inhibitor; PSA: prostate-specific antigen; PSA-DT: prostate-specific antigen doubling time

Variable	Total
Number, n (%)	36
Median age at darolutamide initiation, years (IQR)	77.5 (72.8-83.3)
Median height, cm (IQR)	160.5 (152.3-164.5)
Median weight, kg (IQR)	57.0 (40.5-63.5)
Median BMI, kg/m^2^, (IQR)	21.5 (16.0-23.0)
ECOG PS, n (%)	
0	29 (80.6)
1	4 (11.1)
Missing	3 (8.3)
Gleason grade group at initial diagnosis, n (%)	
1	4 (11.1)
2	4 (11.1)
3	2 (5.6)
4	7 (19.4)
5	17 (47.2)
Missing	2 (5.6)
T stage at initial diagnosis, n (%)	
T2	23 (63.9)
T3a	7 (19.4)
T3b	2 (5.6)
T4	3 (8.3)
Tx	1 (2.8)
History of prior radical treatment, n (%)	
None	10 (27.8)
Surgery	10 (27.8)
Radiation therapy	16 (44.4)
Prior ARSI use, n (%)	5 (13.9)
Median PSA at darolutamide initiation, ng/mL	1.57 (0.48-3.92)
Median PSA-DT at darolutamide initiation, month (IQR)	4.60 (3.0-7.1)
Initial dose of darolutamide, n (%)	
1200 mg	35 (97.2)
600 mg	1 (2.8)
Median follow-up period (IQR)	23.0 (16.0-47.3)

Efficacy of darolutamide

The proportions of patients who achieved ≥50% and ≥90% declines in PSA response were 88.9% and 69.4%, respectively (Figure 1c). Kaplan-Meier analysis showed that both median PFS and OS were not reached (95% CI, 45 months-not available (NA) and 95% CI, NA-NA, respectively) (Figures 1d, 1e). Disease progression occurred in nine patients (25%), prostate cancer-related deaths in two (5.6%), and death from other causes in one (2.8%).

Efficacy was then compared by age-based subgroups using cutoff points of 75 and 80 years. Median PSA at darolutamide initiation, PSA-DT, Gleason grade ≥4, T stage ≥3, and median follow-up period did not differ between the subgroups (Table 2).

**Table 2:** Patients' characteristics stratified by age Comparisons of categorical variables between groups were performed using either the chi-square test or Fisher’s exact test, whereas continuous variables were analyzed with the Mann-Whitney U test. IQR: interquartile range; PSA: prostate-specific antigen; PSA-DT: prostate-specific antigen doubling time

Variable	<75 years group	≥75 years group	P-value	<80 years group	≥80 years group	P-value
Number, n	11	25	-	23	13	-
Median age at darolutamide initiation, years (IQR)	70.0 (65.0-72.0)	80.0 (77.0-85.0)	<0.001	75.0 (70.0-76.5)	85.0 (83.0-87.0)	<0.001
Gleason grade group >4 at initial diagnosis, n (%)	7 (70.0)	10 (41.7)	0.259	12 (54.5)	5 (41.7)	0.721
T stage ≥3 at initial diagnosis, n (%)	4 (36.4)	8 (33.3)	1.000	10 (43.5)	2 (16.7)	0.149
No prior radical treatment, n (%)	8 (72.7)	15 (60.0)	0.708	16 (69.6)	7 (53.8)	0.474
Median PSA at darolutamide initiation, ng/mL	0.45 (0.11-7.70)	1.77 (0.99-3.15)	0.236	0.99 (0.19-4.61)	1.93 (1.50-2.65)	0.205
Median PSA-DT, month (IQR)	4.50 (3.30-6.55)	4.80 (2.88-7.50)	0.859	4.75 (2.95-6.68)	4.60 (3.40-8.90)	0.785
Median follow-up period (IQR)	29.0 (17.0-48.0)	21.0 (16.0-37.0)	0.439	29.0 (16.0-48.0)	21.0 (19.0-31.0)	0.478

**Figure 2:**
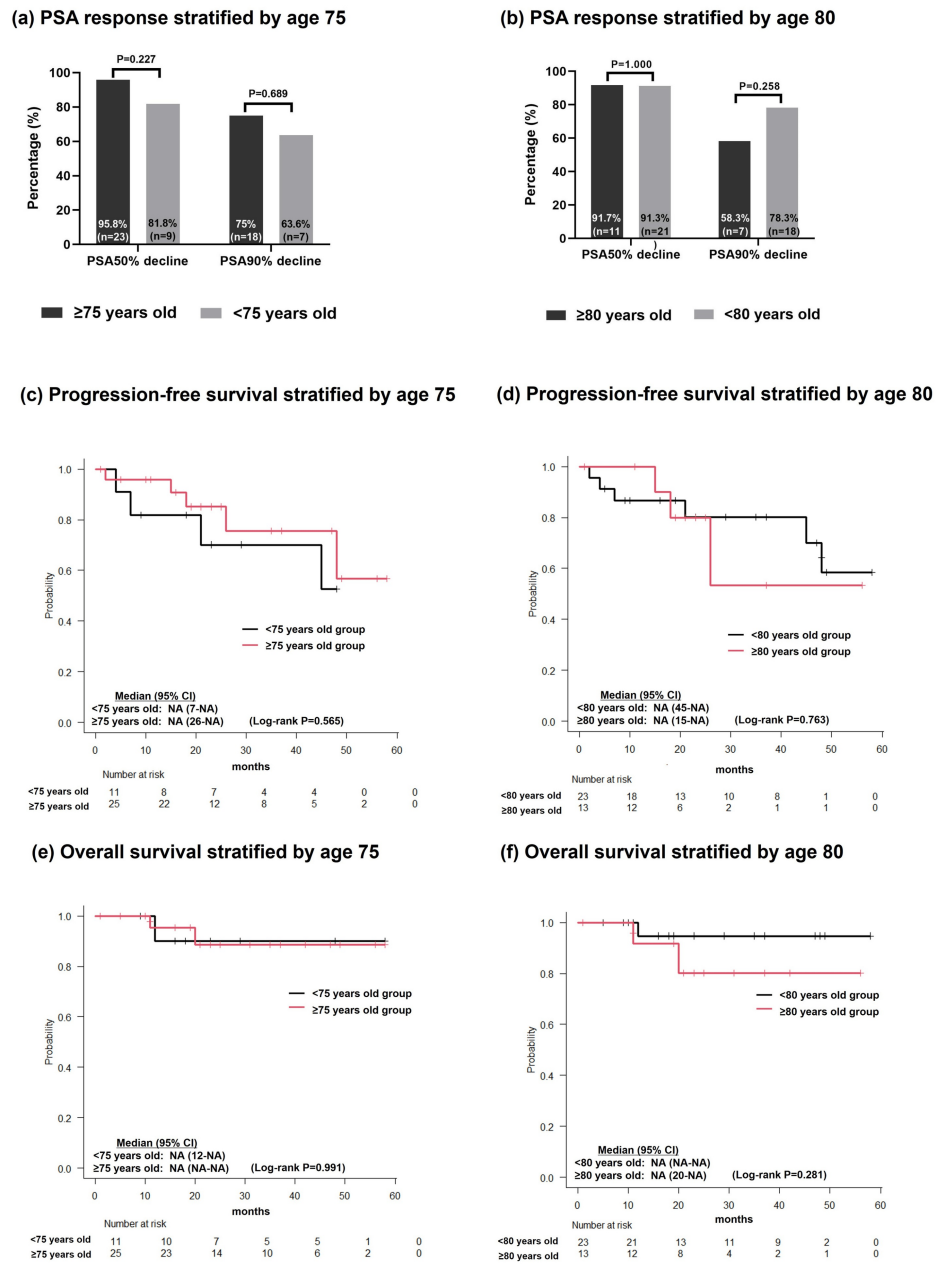
(a) PSA response stratified by age 75, (b) PSA response stratified by age 80, (c) progression-free survival stratified by age 75, (d) progression-free survival stratified by age 80, (e) overall survival stratified by age 75, (f) overall survival stratified by age 80 PSA responses were compared using the chi-square test or Fisher’s exact test. Regarding the Kaplan-Meier method, differences between groups were compared using the log-rank test. PSA: prostate-specific antigen; CI: confidence interval; NA: not available

PSA response did not differ between age subgroups. For the ≥75 vs. <75 years subgroup, ≥50% decline occurred in 95.8% vs. 81.8% (P = 0.227) and ≥90% decline in 75% vs. 63.6% (P = 0.689; Figure 2a). For the ≥80 vs. <80 years subgroup, ≥50% decline occurred in 91.7% vs. 91.3% (P = 1.000) and 90% decline in 58.3% vs. 78.3% (P = 0.258; Figure 2b).

Regarding survival, no significant differences in PFS were observed between the age subgroups. Median PFS was not reached for the ≥75 years (95% CI, 26-NA) vs. <75 years (95% CI, 7-NA) subgroups (log-rank P = 0.565; Figure 2c) and for the ≥80 years (95% CI, 15-NA) and <80 years (95% CI, 45-NA) subgroups (log-rank P = 0.763; Figure 2d). Similarly, OS did not differ significantly, with median OS not achieved in either subgroup (Figures 2e, 2f).

Safety during darolutamide treatment

**Figure 3:**
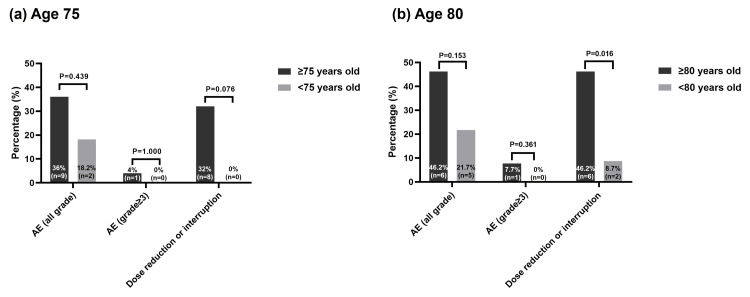
Adverse events (AEs) and darolutamide treatment adjustments stratified by age, (a) age 75, (b) age 80 These comparisons were analyzed using Fisher’s exact test.

Table 3 shows AEs and darolutamide treatment adjustments. Of the 36 men treated with darolutamide, 30.6% (n = 11) experienced AEs of any grade, while 2.8% (n = 1) experienced grade 3 or higher AEs. Grade 1 AEs included loss of appetite, rash, fatigue, liver dysfunction, and hot flash (each n = 1), while grade 2 AEs included nausea, fatigue, anemia, rash, and liver dysfunction (each n = 1). Febrile neutropenia was the only grade 3 or higher AE observed (2.8%, n = 1). Stratification by age subgroups showed no significant differences in all-grade or grade ≥3 AEs in the 75-year threshold subgroup (all-grade: ≥75 years 9/25 (36.0%) vs. <75 years 2/11 (18.2%), P = 0.439; grade ≥3: ≥75 years 1/25 (4.0%) vs. <75 years 0/11 (0.0%), P = 1.000) (Figure 3a). Similar findings were observed for the 80-year threshold subgroup (all-grade: ≥80 years 6/13 (46.2%) vs. <80 years 5/23 (21.7%), P = 0.153; grade ≥3: ≥80 years 1/13 (7.7%) vs. <80 years 0/23 (0.0%), P = 0.361) (Figure 3b). However, patients aged ≥80 years showed significantly higher rates of dose reduction or treatment interruption during darolutamide treatment (6/13 (46.2%) vs. 2/23 (8.7%), P = 0.016) (Figure 3b).

**Table 3:** Adverse events and darolutamide treatment adjustments

Adverse event category	n (%)
Adverse event (all-grade)	11 (30.6)
Adverse event (grade ≥3)	1 (2.8)
Dose reduction or withdrawal	8 (22.2)
Discontinuation due to adverse events	2 (5.6)
Breakdown of adverse events, n (%)	
Grade 1	Loss of appetite (1), rash (1), fatigue (1), liver dysfunction (1), hot flash (1)
Grade 2	Nausea (1), fatigue (1), anemia (1), rash (1), liver dysfunction (1)
Grade 3	Febrile neutropenia (1)

## Discussion

This multi-institutional retrospective study aimed to examine the treatment outcomes of darolutamide in patients with nmCRPC, stratified by age groups (≥75 vs. <75 years and ≥80 vs. <80 years). Our key findings indicated that oncological outcomes, specifically PSA response, PFS, and OS, were comparable between older and younger patient groups. Additionally, the overall incidence of AEs was similar across age groups. However, a significant finding was the higher rate of darolutamide dose reduction or treatment interruption observed in patients aged ≥80 years. This study highlights several critical aspects of darolutamide use in real-world clinical practice for men with nmCRPC.

First, the consistent PSA response and PFS between older and younger patients suggest that darolutamide can provide good disease control regardless of age. These results are in line with the findings of a post-hoc analysis of the ARAMIS trial [13]. The subgroup analysis in this trial was focused on vulnerable populations based on the number of comorbidities and concomitant medications. Patients with multiple comorbidities and patients using multiple concomitant medications were typically aged 75 years or older. The analysis showed that the OS benefit of darolutamide versus placebo remained consistent in such vulnerable populations [13]. These results support the conclusion that darolutamide maintains a favorable benefit/risk ratio, significantly improving oncological outcomes without diminished effectiveness in medically complex, older patients.

Second, no significant differences were observed in the incidence of all-grade or grade ≥3 AEs between older patients (aged ≥75 and ≥80 years) and younger patients receiving darolutamide. This finding suggests that the safety profile of darolutamide remains favorable in elderly patients and is consistent with previously reported results from the phase III ARAMIS trial [3,7], in which the median age of participants was approximately 74 years. In the darolutamide group, the incidence of AEs was similar to that in the placebo group, and treatment-discontinuing AEs occurred at a low rate of approximately 9% in both groups [7]. Darolutamide is recognized for its favorable safety profile, mainly due to its low blood-brain barrier penetration [6]. This characteristic contributes to a low incidence of CNS-related AEs, with rates in the ARAMIS trial being similar to those of placebo [7]. There were no fatal AEs in the present study. Only one case of grade 3 AE (febrile neutropenia) occurred on day 25 of darolutamide administration (Table 2). This condition improved with antibiotic therapy and administration of granulocyte colony-stimulating factor. Darolutamide was restarted at a reduced dose but was discontinued owing to fatigue.

On the other hand, dose reductions or treatment interruptions occurred significantly more frequently in patients aged ≥80 years compared with younger patients (46.2% vs. 8.7%, P = 0.016) (Figure 3b). This trend suggests that, even in the absence of grade ≥3 AEs, physicians may adopt a more cautious approach to treatment management in elderly patients. In clinical practice, older patients often present with frailty, cognitive decline, and impaired activities of daily living, which can render even grade 1-2 AEs impactful on functional status and quality of life. Consequently, physicians may perform dose reductions or temporary treatment interruptions. In addition, elderly patients often experience multiple comorbidities and polypharmacy, necessitating careful consideration of potential drug-drug interactions [14]. Darolutamide has a lower potential for drug interactions, which means that dose adjustments are less frequently required, thereby reducing the likelihood of increased AE risk. In fact, a large-scale retrospective cohort study in the United States revealed that treatment discontinuation rates were significantly lower with darolutamide than with enzalutamide or apalutamide, suggesting superior tolerability [8]. Such favorable real-world tolerability of darolutamide may offer advantages for treatment continuation in elderly patients. Nonetheless, the fact that approximately half of patients aged ≥80 years in our analysis required dose adjustments indicates that, in the very elderly population, careful consideration of the balance between treatment efficacy and AEs risk is essential, and meticulous monitoring may be warranted.

The present study has some limitations. First, the number of patients was limited. Our subgroup analyses were based on relatively small strata (≥75 vs. <75 years: 25 vs. 11; ≥80 vs. <80 years: 13 vs. 23), which reduces statistical power. They should be interpreted with caution, given the small, retrospective cohort. Therefore, further research is required in larger-scale settings. Second, due to its multi-institutional retrospective design, potential biases from patient selection, the lack of centralized imaging review, and variations in data collection, including the profiles of AEs, could be present across institutions. Consequently, the real-world clinical data analyzed in this study were derived from a context that differed from that of clinical trials. Additionally, the evolving landscape of imaging technologies, such as prostate-specific membrane antigen, positron emission tomography/computed tomography, may redefine nmCRPC in the future [15]. The dependence on conventional imaging criteria for defining nmCRPC in the present study, while consistent with that of the ARAMIS trial, may not fully capture the range of lesions detectable with new modalities.

## Conclusions

Our findings indicate that darolutamide demonstrates consistent efficacy across various age groups in patients with nmCRPC. While the overall safety profiles were similar, patients aged ≥80 years more frequently required dose adjustments or temporary treatment interruptions (46.2% vs. 8.7%, P = 0.016). These findings support the use of darolutamide in elderly patients, providing practical guidance for managing older patients with nmCRPC in clinical practice, such as early, close monitoring for tolerability and shared decision-making regarding treatment goals.

## References

[1] Scher HI, Solo K, Valant J, Todd MB, Mehra M (2015). Prevalence of prostate cancer clinical states and mortality in the United States: estimates using a dynamic progression model. PLoS One.

[2] Fizazi K, Shore N, Tammela TL (2019). Darolutamide in nonmetastatic, castration-resistant prostate cancer. N Engl J Med.

[3] Fizazi K, Shore N, Tammela TL (2020). Nonmetastatic, castration-resistant prostate cancer and survival with darolutamide. N Engl J Med.

[4] Smith MR, Saad F, Chowdhury S (2021). Apalutamide and overall survival in prostate cancer. Eur Urol.

[5] Hussain M, Fizazi K, Saad F (2018). Enzalutamide in men with nonmetastatic, castration-resistant prostate cancer. N Engl J Med.

[6] Williams SC, Mazibuko N, O'Daly O (2023). Comparison of cerebral blood flow in regions relevant to cognition after enzalutamide, darolutamide, and placebo in healthy volunteers: a randomized crossover trial. Target Oncol.

[7] Shore ND, Gratzke C, Feyerabend S (2024). Extended safety and tolerability of darolutamide for nonmetastatic castration-resistant prostate cancer and adverse event time course in ARAMIS. Oncologist.

[8] George DJ, Morgans AK, Constantinovici N (2024). Androgen receptor inhibitors in patients with nonmetastatic castration-resistant prostate cancer. JAMA Netw Open.

[9] Fujiwara R, Yamamoto S, Takemura K (2024). Clinical outcomes and prognostic factors in nonmetastatic castration-resistant prostate cancer treated with androgen receptor signaling inhibitors therapy. Cancers (Basel).

[10] Scher HI, Halabi S, Tannock I (2008). Design and end points of clinical trials for patients with progressive prostate cancer and castrate levels of testosterone: recommendations of the Prostate Cancer Clinical Trials Working Group. J Clin Oncol.

[11] Tohi Y, Kobayashi K, Daizumoto K (2025). Real-world clinical usage and efficacy of apalutamide in men with nonmetastatic castration-resistant prostate cancer: a multi-institutional study in the CsJUC. Jpn J Clin Oncol.

[12] Kanda Y (2013). Investigation of the freely available easy-to-use software 'EZR' for medical statistics. Bone Marrow Transplant.

[13] Fizazi K, Shore ND, Smith M (2023). Efficacy and safety outcomes of darolutamide in patients with non-metastatic castration-resistant prostate cancer with comorbidities and concomitant medications from the randomised phase 3 ARAMIS trial. Eur J Cancer.

[14] Ibáñez C, Tourís-Lores M, Montesa Á (2025). Drug-drug interactions in metastatic hormone-sensitive prostate cancer (mHSPC): practical considerations for treating men with androgen receptor pathway inhibitors and common medications in this stage. Expert Opin Drug Metab Toxicol.

[15] Fendler WP, Weber M, Iravani A (2019). Prostate-specific membrane antigen ligand positron emission tomography in men with nonmetastatic castration-resistant prostate cancer. Clin Cancer Res.

